# Consumer perception of attributes of organic food in Italy: A CUB model study

**DOI:** 10.1016/j.heliyon.2022.e09007

**Published:** 2022-02-25

**Authors:** Emilia Lamonaca, Barbara Cafarelli, Crescenza Calculli, Caterina Tricase

**Affiliations:** a Department of Sciences of Agriculture, Food Natural Resources and Engineering, University of Foggia, Via Napoli 25, 71121 Foggia, Italy; b Department of Economics, Managment and Territory, University of Foggia, Via da Zara I, 71121 Foggia, Italy; c Department of Economics and Finance, University of Bari Aldo Moro, Largo Abbazia S. Scolastica, 70124 Bari, Italy; d Department of Economics, University of Foggia, Largo Papa Giovanni Paolo II, 71121 Foggia, Italy

**Keywords:** Healthiness, Food safety, Sustainability, Organic food, Consumer, CUB models

## Abstract

Organic food, consumers and their buying behaviour are well examined fields of research, although there is a lack of consistent findings on consumers' perception about organic food's quality, in terms of healthiness, safety, and environmental sustainability, and on determinants of perceived quality. This study investigates how consumers perceive the quality of organic food, in terms of environmental sustainability, safety, and healthiness. The study also analyses how and to what extent perceived quality of organic food is influenced by the presence of information related to quality on food products' labels and consumers' socio-demographic profile. A survey has been conducted on a convenience sample of Italian consumers, recruited through a snowball sampling technique. An approach based on a Combination of Uniform and shifted Binomial random variables, named CUB model, is adopted to analyse consumers' perceptions in terms of two latent components, feeling and uncertainty. The CUB model approach is suitable for analyses that involve consumers perception. The results suggest that consumers perceive safety of organic food better than healthiness and environmentally sustainable attributes. Findings also highlight that the presence of specific information on food's label contributes to perceive organic food as healthier, safe, and environmentally sustainable: the more the details on food labels, the higher the consumers' perception. Furthermore, consumers' socio-demographic profile plays a significant role: males and females have a different perception of organic food and younger consumers tend to be more prone to buy and consume organic product.

## Highlights

• Consumers are more confident with healthiness and sustainability of organic food.
• Males and females have a different perception of organic food.
• Food labels increase the perception of organic food as healthy, safe, sustainable.
• More details on labels of organic food enhance consumers' perception.

## 1 Introduction

### 1.1 Background and motivation

During the last decades, a major challenge of the agri-food sector is to be sustainable, from environmental, social, and economic perspectives. Several farms and firms operating in the agri-food sector have introduced principles of environmental, social, and economic sustainability into their business models (Migliore et al., 2015a; Schimmenti et al., 2016). The growing orientation towards the three pillars of sustainability (i.e., environmental, social, and economic) is mostly driven by a change in consumer behaviours due to the increasing awareness for the environmental and social attributes of products they consume (Santeramo et al., 2018; Galati et al., 2019). In fact, the increasing demand for food, driven by a growing population, is exerting high pressure on land and production of inputs, causing detrimental impacts for human and environmental wellbeing (Tricase et al., 2018; Yanakittkul and Aungvaravong, 2020; Santeramo et al., 2021). The production and consumption of food should avoid adverse effects both for human (e.g., unhealthy diets) and environmental (e.g., climate changes, resources depletion) welfare (Lazzarini et al., 2016; Santeramo et al., 2020a, Santeramo et al., 2020b). The progressive shift towards responsible behaviours, observed in the production and consumption of organic food, moves in this direction (Dowd and Burke, 2013; Lamonaca et al., 2016). The latest available data from the world of organic agriculture (Willer et al., 2021a, Willer et al., 2021b) show that, in 2019, 72.3 million hectares of farmland were organic worldwide. Organic farmland increased by seven-fold in twenty years and organic producers (3.1 million in 2019), processors (over 105,000 in 2019), and importers (approximately 7,300 in 2019) are on the rise. Europe had the largest area under organic agricultural management (i.e., 16.5 million hectares, 3.3% of its total agricultural land), after Oceania, and a relevant growth has been observed in the last decade (+60.8%) and particularly compared to the previous year (+5.9%). With 2.0 million hectares (15.2% of its total agricultural land), Italy was among the top 10 countries worldwide and the third country in Europe with the largest areas of organic agricultural land. The organic land increased by + 1.8% with respect to the previous year and by +79.0% from 2010 to 2019 in Italy. These trends are comforting and go towards the achievement of one of the targets set by the European Commission in the Farm to Fork strategy: i.e., “reaching at least 25% of the EU agricultural land under organic farming by 2030” (European Commission, 2020). While a continued growth is observed in all key indicators of the organic sector, the European organic market grew more than the organic area. Europe accounted for more than 430,000 producers, 78,000 processors, and 6,500 importers: compared to 2018, the number of importers (+12.1%) grew faster than the number of producers (+2.8%) and processors (+8.5%). Italy was among the 10 countries with the most organic producers (i.e., 70,561) and the country with the largest number of processors (i.e., 21,940). Organic retail sales in Europe were valued at 45.0 billion euro and reached 3,625 million euro in Italy, one of the leading countries in terms of shares of organic market (3% worldwide and 9% in Europe). Italian consumers spent 60 euro on organic food per person, more than the average per capita consumption in Europe (i.e., 56 euro). This strong market growth is continuing the trend of the past several years (Travnicek et al., 2021).

Several definitions have been proposed for the organic agriculture. The Food and Agriculture Organisation (FAO) Conference on Organic Agriculture and Food Security in 2007 defined it as a neotraditional food system. In 2008, the General Assembly of the International Federation of Organic Agriculture Movements (IFOAM)^1^ defined organic agriculture as “*a production system that sustains the health of soils, ecosystems, and people. It relies on ecological processes, biodiversity and cycles adapted to local conditions, rather than the use of inputs with adverse effects. Organic Agriculture combines tradition, innovation, and science to benefit the shared environment and promote fair relationships and good quality of life for all involved*” (IFOAM - International Federation of Organic Agriculture Movements, 2018). Similarly, the FAO Glossary on Organic Agriculture^2^ (p. 99) defines organic agriculture as “*a holistic production management system which promotes and enhances agroecosystem health, including biodiversity, biological cycles, and soil biological activity. It emphasizes the use of management practices in preference to the use of off-farm inputs, taking into account that regional conditions require locally adapted systems. This is accomplished by using, where possible, cultural, biological and mechanical methods, as opposed to using synthetic materials, to fulfil any specific function within the system.*”. Accordingly, section 2.1 of the Codex Alimentarius^3^ clarifies that “*Foods should only refer to organic production methods if they come from an organic farm system employing management practices which seek to nurture ecosystems which achieve sustainable productivity, and provide weed, pest and disease control through a diverse mix of mutually dependent life forms, recycling plant and animal residues, crop selection and rotation, water management, tillage and cultivation.*”. All these definitions agree in considering the organic agriculture as a socially, ecologically, and economically sustainable production systems.

Organic markets are growing but reactive, driven by food safety concerns and environmental awareness (FAO Glossary on Organic Agriculture, p. 100). In fact, organic products have unobservable characteristics to which consumers attach a high value (Nedra et al., 2015; Vrontis et al., 2015): the growing demand for organic food is driven by the perception of consuming healthy, safe, and sustainable food (e.g., Aschemann-Witzel et al., 2013; Bryla, 2016; Schrank and Running, 2018). Major criticisms towards conventional food are related to the issues of intensive agricultural practices (e.g., Hsu and Chan, 2014), residues in food from synthetic pesticides and fertilisers (e.g., Michaelidou and Hassan, 2008; Hwang, 2016) and presence of Genetically Modified Organism (GMO) (e.g., Yadav and Pathak, 2016). The Codex Alimentarius clarifies that organic production and processing methods require that for the production and preparation of organic products a set of production and processing requirements (i.e., the ones listed in the Annex 1 of the Codex – “*Principles of organic production*”) should be satisfied. For instance, the recycling of plant nutrients is a fertilising strategy adopted to ensure sustainability in the organic production methods. The biological and cultural control and mechanical removal of pests or the use of beneficial insect populations are management strategies that should be adopted to prevent pests and diseases in the organic crop production. Similarly, the prevention of diseases in the organic animal production should be based, for instance, on the provision of good quality organically grown feedstuffs and animal management practices, while avoiding the use of antibiotics and other chemical allopathic veterinary drugs. The rules of production and preparation set forth in the Codex Alimentarius (i.e., Section 4 and Annex 1) are resumed and widened by the Regulation (EC) No. 834/2007 of European Union which lays down the legislative framework relevant to the sustainable development of the European organic markets. The legislative framework sets forth rules applicable to processed and unprocessed products originating from organic agriculture in the European Union. They concern all stages of production, preparation and distribution of organic products, and the use of the organic indications on products' labelling. Important revisions are ongoing in the legislation of the organic market in the European Union (Kirchner et al., 2021; Willer et al., 2021a, Willer et al., 2021b). The new organic Regulation (EU) No. 848/2018, entered into force on the 1st of January 2022, revise and strengthen the existing legislation on the production and labelling of organic products, the Regulation (EC) No 834/2007. The regulation broadens the scope of the European Union's rules in terms of control system, trade regime, and production rules concerning the organic market. One of the key points of the new regulation is the harmonisation and the simplification of the rules applicable to organic operators in the EU Member States and non-EU countries through the introduction of the compliance system. Indeed, the compliance with multiple standards, certification requirements, and regulations is one of the main obstacles for the development of the organic sector. According to the International Task Force on Harmonization and Equivalence in Organic Agriculture^4^, two international standards for organic agriculture (i.e., Codex Alimentarius Commission Guidelines, IFOAM Basic Standards), hundreds of private sector standards and governmental regulations, many certification and accreditation systems exist in the organic market and the mutual recognition and equivalency among them are limited.

### 1.2 Previous studies and gap in literature

The literature on consumer decision-making process highlights the crucial role of the consumers' preferences for high-quality products (e.g., Galati et al., 2015; Santeramo et al., 2020a, Santeramo et al., 2020b; Santeramo and Lamonaca, 2020a, Santeramo and Lamonaca, 2020b, 2021). Quality and credence attributes, such as environmental sustainability and healthiness, are determinant in guiding consumers' buying behaviours (Migliore et al., 2015b). Some studies demonstrate that health and safety concerns are main reasons that lead consumers to choose organic food (e.g., Magnusson et al., 2003; Ghvanidze et al., 2016; Lazzarini et al., 2016; Prada et al., 2016), others conclude on the importance of environmental welfare as driver of consumers’ choice of organic food (e.g., Padel and Foster, 2005; Mondelaers et al., 2009; Zander and Hamm, 2010; Lee and Yun, 2015; Moser, 2016).

Although these motivations (i.e., health and environmental concerns) tend to have a strong direct and positive effect on the buying behaviour of organic products (Nedra et al., 2015; Vrontis et al., 2015), declared preferences often do not translate into real product purchases (Galati et al., 2019). These studies demonstrate that, although consumers declare their preferences for organic food due to their environmental sustainability, the healthiness of organic products is the attribute that guide consumers’ buying behaviour.

Previous studies also suggest that consumers' perception of organic food is largely influenced by consumers' characteristics (e.g., Hsu et al., 2012; Hsu and Chen, 2014) and by the presence of specific labels (Prada et al., 2016). Recently, Migliore et al. (2020) show that attitudes towards healthy eating and the environment are positively associated with a higher willingness to pay for organic products, the latter being also affected by consumers' socio-demographic characteristics. Some studies demonstrate that consumers' perception is affected by health claims and nutrition information on packaging (Ghvanidze et al., 2016), climate friendliness through a carbon footprint label (Meyerding, 2016), organic and quality labels (Lazzarini et al., 2016). For instance, Galati et al. (2019) argue that conscious consumers pay more attention to information on labels and conclude that such information positively affect consumers’ willingness to pay for organic wine.

Although organic food consumers study is a well examined fields, it is still not clear the linkage among healthiness, safety, and environmental sustainability of organic food. According to the definitions proposed by the European Food Safety Authority (EFSA)^5^, safety is related to a food considered safe, for which an adverse effect is unlikely to occur. The evaluation of safety for a certain food depends on the potential toxic effect associated with the consumption of that food, and on the size and type of the population consuming that food to be protected from potential toxic effects. Healthiness is the characteristic of a certain food whose consumption is likely to produce health benefits due to particular nutrients or ingredients contained in that food. Environmental sustainability concerns a food obtained avoiding the use of substances or activities that may harm the environment. The substances or activities may include the use of chemicals and pesticides, the introduction of genetically modified plants and organisms, the spread of pests and diseases. For the organic food, these characteristics are frequently analysed separately. Vice-versa, one of the scopes of organic production is to provide healthful and safe food in a sustainable way (IFOAM - International Federation of Organic Agriculture Movements, 2021). Organic agriculture relies on four principles (i.e., health, ecology, fairness, and care) that evoke the characteristics of healthiness, safety, and environmental sustainability of organic food. The FAO Glossary on Organic Agriculture (p. 101) clarifies that “*Principles apply to agriculture in the broadest sense, including the way people tend soils, water, plants and animals in order to produce, prepare and distribute goods. They concern the way people interact with living landscapes, relate to one another and shape the legacy of future generations. Each principle is followed by an action-oriented explanation.*”. According to the principle of health, “*organic agriculture should sustain and enhance the health of soil, plant, animal, human and planet as one and indivisible*” (IFOAM - International Federation of Organic Agriculture Movements, 2021). Thus, organic food involves the concepts of healthiness and environmental sustainability: a healthy environment allows to produce healthy food contributing to maintain human, animal, and plant well-being. Coherently, the use of chemicals (e.g., fertilisers, pesticides, animal drugs, food additives), dangerous for both health and environmental sustainability, and of risky technologies, potentially jeopardising food safety, should be avoided in the production of organic food. As stated in the principle of care “*organic agriculture should be managed in a precautionary and responsible manner to protect the health and well-being of current and future generation and the environment*” (IFOAM - International Federation of Organic Agriculture Movements, 2021). Precautionary and responsible behaviours of stakeholders and policy makers require the assessment of new production practices and technologies and the revision of existing production methods to ensure that organic food is healthy, safe, and environmentally sustainable. Indeed, the production of organic food should “*be based on ecological processes and recycling*” and “*ensure fairness with regard to the common environment and life opportunities*”, as required by the principles of ecology and fairness (IFOAM - International Federation of Organic Agriculture Movements, 2021).

### 1.3 Contribution to the existing knowledge

This study investigates consumers' perception of three attributes of organic food: i.e., healthiness, safety, environmental sustainability. These three attributes are derived from the from the principles on which organic agriculture grows and develops. Food from organic agriculture should be healthy and of high quality to contribute maintaining physical, mental, social, and ecological well-being (principle of health). Organic food is safe to the extent that it is produced by adopting appropriate technologies that prevent significant risks (principle of care): for instance, organic food should be produced avoiding genetic engineering. Resources that are used for production and consumption of organic food should be managed in a socially and environmentally sustainable way (principle of fairness) to achieve the ecological balance and ensure the protection of the environment (principle of ecology). The study also examines to what extent the perception of these attributes is influenced by consumers' socio-demographic profile and by the presence of specific labels. The use of CUB models is motivated by the purpose of this research: i.e., investigating how consumers perceive the quality of organic food^6^. Specifically, CUB models allow to estimate consumers’ perception (i.e., sentiment) for organic food (i.e., attribute of interest) using only the rating that the consumer attributes to a certain statement/item expressed in a Likert scale with at least three levels. Furthermore, these models combine the estimation of feeling towards a statement with an estimation of uncertainty depending on the ability of the respondent to translate his sentiment into a rating. It is worth to highlight that CUB model allows to estimate the feeling and the uncertainty separately by considering a mixture of two random distributions.

Many empirical evidence and methodological studies refer to this model-based approach as an effective and consolidated statistical framework (Capecchi and Simone, 2019). The proposed models allow to directly formulate the probability distribution of ordinal/discrete data and to mimic the psychological mechanism below the perception expression. The CUB models consider the decision-making process of the observed score as the sum of two components: i.e., the personal feeling/liking of consumers toward product characteristic and the uncertainty over consumers ability to transfer perceptions into hedonistic scales in an ordinal value (Piccolo 2003; D'Elia and Piccolo 2005; Piccolo and D'Elia 2008). These models provide a measure of consumers' feeling/liking for a product together with a measure of intrinsic heterogeneity which is linked to the consumer ability to rank the evaluation (D'Elia and Piccolo 2005). In this way, CUB models are useful for modelling the stochastic structure of judgment evaluation process and for adequately representing observed perceptions (Grilli et al., 2014; Carpita et al., 2019). In this model-based approach, the propensity of a meditated choice is formally described by a shifted Binomial random variable whereas a totally random choice is described by a discrete Uniform distribution. As a consequence, CUB models allow to measure the respondents' attitude by means of this mixture distribution.

## 2 Methodological framework

An online survey has been conducted on a convenience sample of Italian consumers. The respondents were requested to judge some aspects/items by expressing their level of agreement about statements by using a Likert type scale. The collected rating data are analysed using an approach based on CUB models (Piccolo, 2003), a Combination of Uniform and shifted Binomial random variables which is suitable to model consumers' perceptions (Piccolo and D'Elia, 2008). The CUB models are useful for the analysis of ordinal data arising from customer satisfaction surveys, consumer tests, market segmentation and product positioning (Piccolo and D'Elia 2008; Iannario et al., 2012; Corduas et al., 2013; Capecchi et al., 2018; Punzo et al., 2018).

### 2.1 Data collection and methods

An online survey was carried out in order to analyse the perception of organic food for a sample of Italian consumers. Data has been collected through a questionnaire based on a review of the literature on the issue (Table 1). The items of the questionnaire have been selected considering the most addressed topics in the literature. Healthiness, safety, and environmental sustainability of organic food are attributes traditionally investigated in consumers studies (e.g., Mohd Suki, 2015; Seegebarth et al., 2016; Migliore et al., 2020). Similarly, the contribution of specific food labels on the perception of organic food is the focus of several research (e.g., Ghvanidze et al., 2016; Lazzarini et al., 2016; Meyerding, 2016). The novelty of the structure of our questionnaire is in the joint investigation of these aspects. The validation protocol of the questionnaire has consisted in two steps. First a pilot survey was conducted to skim the items selected from the literature review. Second, the revised questionnaire was preliminary tested among selected respondents.

**Table 1:** Relevant questionnaire items and references.

Item		Scale	References
Attributes of organic food	Organic food is healthier	7-point Likert scale	Seegebarth et al. (2016)
	Organic food is safe	7-point Likert scale	Seegebarth et al. (2016)
	Organic food is environmentally sustainable	7-point Likert scale	Mohd Suki (2015)
Labels	Label info (e.g. facts table, GMO^a^-free, 100% organic)	7-point Likert scale	Carlucci et al. (2017)
	Health claims (e.g. Organic foods are not necessarily completely chemical free, but the pesticide residues will be considerably lower than those found in produce manufactured with synthetic chemicals)	7-point Likert scale	Ghvanidze et al. (2016)
	Quality label (e.g. PDO^b^, PGI^c^)	7-point Likert scale	Lazzarini et al. (2016)
	Organic label	7-point Likert scale	Lazzarini et al. (2016)
	Environmental label (e.g. Ecolabel)	7-point Likert scale	Meyerding (2016)

a Genetically Modified Organism (GMO).

b Protected Designation of Origin (PDO).

c Protected Geographical Indication (PGI).

A pilot survey suggests that respondents are reluctant to provide details on income levels, due to the sensitive nature of this information. Accordingly, we collect only information on the weekly spending for food. The questionnaire (available in Appendix) is as short as possible to avoid a high abandonment rate of respondents: it only took a few minutes (less than 10) to be filled.

The questionnaire consists of 7 questions (in 13 items) divided into three sections. The first one allows for some socio-demographic information. The second section investigates the respondents' perception of organic food as healthier, safe, and environmentally sustainable. The third one examines the respondents’ perception about the presence of specific information related to food products (label info, health claims, quality label, organic label, environmental label).

The questions in sections 2 and 3, split in different items, are measured using a 7-point agree/disagree Likert scale, where 1 represents an extremely negative and 7 indicates a completely positive judgment (Table 1).

The questionnaire, preliminary tested among selected respondents, was available from May to July 2018 on Google Forms. Respondents were recruited through invitations to participate in the online survey via social networks (e.g., Facebook, LinkedIn) and e-mail lists (e.g., academic community, external collaborators, personal contacts). In order to reach large number of respondents, snowball sampling recruitment was adopted using interpersonal relations and connections among respondents. This sampling technique is consistent with previous studies aimed at profiling organic consumers (e.g., Galati et al., 2019; Migliore et al., 2020). This online survey method was chosen since it is an efficient technique to collect a complex set of information in a reasonably short period of time (McCullough, 1998) and it is suitable for the application of CUB models. The final sample consists of 672 respondents, a suitable number considering that more than 10 cases per parameter are attributable to each item (D'Elia, 2003). Informed consent was obtained from all respondents and the study complies with all ethical regulations.

Our typical respondent is a woman between 26 and 45 years old with an upper secondary school or higher degree, with an average weekly spending for food between 50.00 € and 150.00 €. Table 2 summarises the demographic profile of respondents in the sample.

**Table 2:** Socio-demographic characteristics of respondents.

Socio-demographic characteristics	N	%
*Gender*		
Male	242	36.0
Female	430	64.0
*Age*		
18–25	128	19.0
26–35	247	36.8
36–45	149	22.2
46–55	98	14.6
More than 55	50	7.4
*Educational level*		
Primary school	4	0.6
Middle school	31	4.6
Upper secondary school	210	31.3
Bachelor/Master's degree or equivalent	427	63.5
*Financial situation*		
Difficult	38	5.7
Modest	154	22.9
Discreet	290	43.2
Good	178	26.5
Very good	12	1.8
*Weekly spending for food*		
Lesser than €50	96	14.3
€50-€100	290	43.2
€100-€150	178	26.5
€150-€200	73	10.9
More than €200	35	5.2

**Figure 1:**
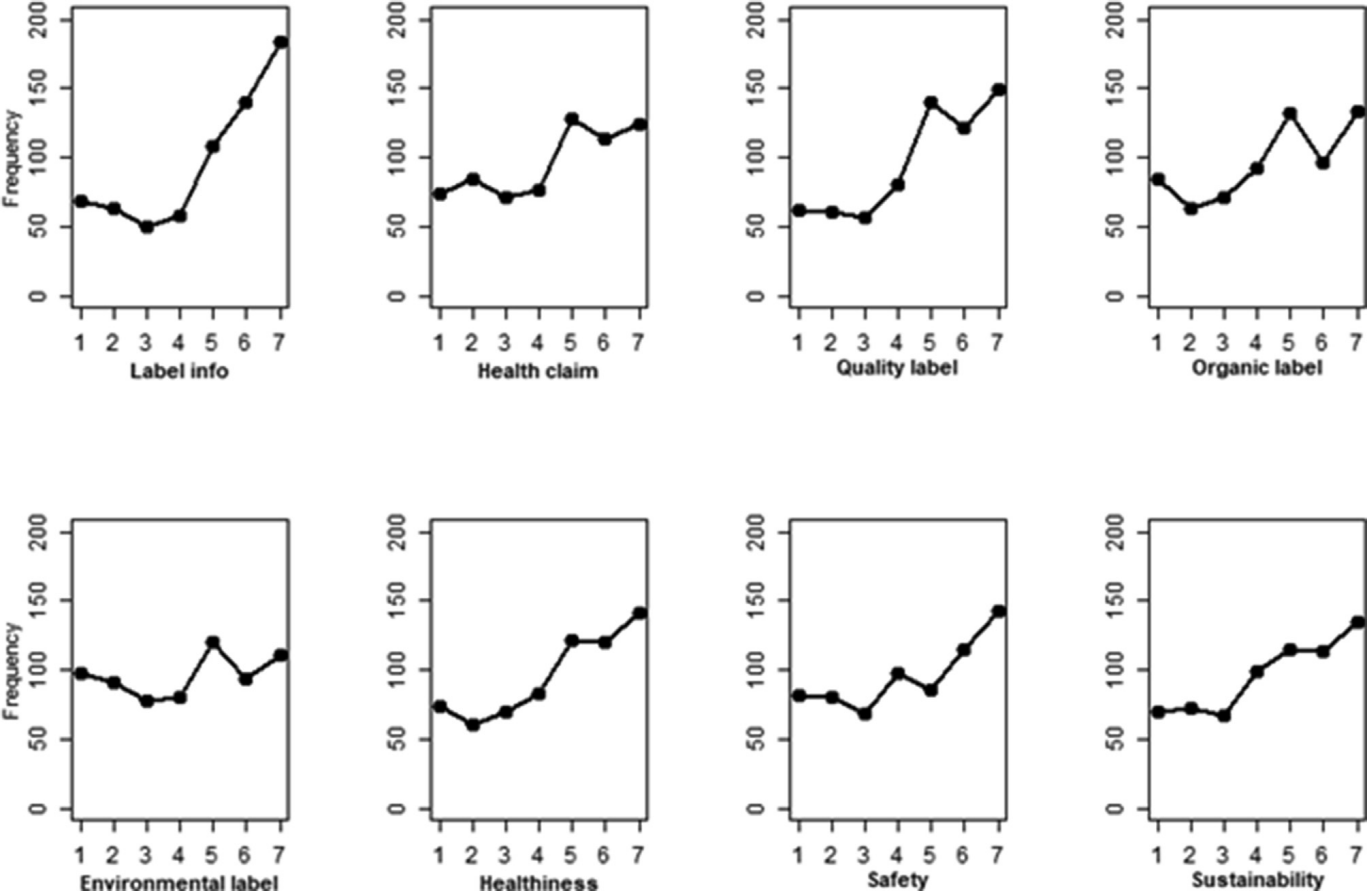
Frequency distributions of relevant items.

The frequency distributions of relevant items in the questionnaire (sections 2 and 3) are shown in Figure 1. The presence of detailed information on label (‘label info’) is skewed towards high rating, considering a 7-point Likert scale of disliking/liking.

### 2.2 The CUB models

The collected rating data are analysed using a probabilistic approach based on CUB models specified as a Combination of Uniform and shifted Binomial random variables (Piccolo, 2003). These models are suitable to model consumers' perceptions (Piccolo and D'Elia, 2008) and analyse perception data sets (Cafarelli et al., 2015; Cafarelli and Crocetta 2016; Capecchi et al., 2016; Iannario and Piccolo, 2016a, Iannario and Piccolo, 2016b). They also represent an advantageous alternative for analysing rating data (Piccolo et al., 2019). The rationale for CUB models (Piccolo, 2003; D'Elia and Piccolo, 2005) is based on the fact that the response of a rater to an item is a weighted combination of two factors, that is a subjective agreement or feeling towards the item and some intrinsic fuzziness or uncertainty in the final response. The first factor is related to awareness of the topic, previous experience, group membership, and so on, whereas the second component results from different facts such as the amount of time available to respond, the use of limited set of information, partial understanding, laziness and so on (Golia, 2015). In this perspective, the CUB model mimics the psychological mechanism that leads a respondent to give an assessment of a certain item and the final judgment is the result from two acting forces: the selectiveness/feeling which represents the intimate attitude that the subject has towards the object under judgment, and the uncertainty due to the fact that the respondent has to convey his personal belief about such object into a single grade of a given scale of measurement (both quantitative or qualitative). These two combined components produce the final score as specified in Section 2.3.1. The use of this model-based approach requires only that respondents/consumers express their judgments on a Likert scale with a certain number of ratings, thus questions related to the reaction response or concerning how sure the respondents feel are not necessary.

To take into account all these aspects and to investigate potential drivers of decisions, the proposed statistical approach that models the respondents' choice mechanism conveys, in a parametric setting, the role of the subjects' covariates and easily allows to handle some inferential issues related to the efficient and consistent maximum likelihood (ML) model parameters estimation and to the minimum sample size required for unbiased parameter estimates (D'Elia, 2003).

#### 2.2.1 The CUB(0,0) models

According to D'Elia and Piccolo (2005), in the evaluation process of a product/service, the subject's judgement may be explained in terms of two latent components: feeling and uncertainty. Feeling is the personal level of liking/disliking for an item under judgment. Uncertainty is the indecision associated with the conversion of perceptions into ordinal values, inherent in the elicitation process and dependent on several circumstances that vary on case-by-case basis. A shifted Binomial random variable and a discrete Uniform random variable are suitable to model, respectively, feeling and uncertainty (Piccolo, 2003). Accordingly, in the CUB models, the ordinal response (rating, r) is the realisation of a discrete random variable (R) with a probability function specified as described in Eq. (1):

(1) $$\mathrm{Pr}\left(R=r\right)=\pi \left(\begin{array}{l} m-1\\ r-1 \end{array}\right)\xi^{m-r}{\left(1-\xi \right)}^{r-1}+\left(1-\pi \right)\left(\frac{1}{m}\right),\;r=1,2,...,m$$

where m is the number of categories of the evaluation scale, ξ and π are the parameters related to feeling and uncertainty.

As discussed in Iannario (2010), the CUB model in Eq. (1) is fully identifiable for any m>3, and defined for ξ∈[0,1] and π∈(0,1]. If ξ<0.5(>0.5), the probability distribution of R is negatively (positively) skewed, with respect to the midpoint $\frac{\left(m+1\right)}{2}$, suggesting that respondents choose their ratings from the end (beginning) of the evaluation scale (Iannario et al., 2012). If π→0 (π→1), R tends to behave as a discrete Uniform (shifted Binomial) distribution, suggesting a completely random (thoughtful) choice (Iannario and Piccolo, 2013). In our case, ($1-\hat{\xi }$) is used to estimate the feeling and ($1-\hat{\pi }$) is used to estimate the uncertainty of respondents. In this perspective, CUB models allow to estimate the choice of ordinal scores by a mixture distribution formally described by shifted Binomial and discrete Uniform random variables. The first one represents the propensity to adhere to a meditated choice and it is the result of a counting process within a sequential selection among the *m* ratings whereas the second one represents the most unpredictable case among all discrete ratings to mimic a pure random choice. It should be noted that for estimating the model parameters related to feeling and uncertainty, it is sufficient that respondents express their judgments on a Likert scale with a number of ratings greater than 3 and that questions related to the reaction response or questions about how sure the respondents feel are not necessary (Piccolo and Simone, 2019). CUB model is in fact fully identifiable for any m > 3 as proved by Iannario (2010) and is well defined for parameters $\theta ={\left(\pi ,\xi \right)}^{\prime }$ .The constraint m>3 avoids considering degenerate (m=1), indeterminate (m=2) or saturated (m=3) models, respectively. Then, we will define as admissible a CUB model such that m>3.

The goodness of fit of the estimated model may be assessed by comparing the observed frequencies ($f_{r}$) and the expected probabilities (${\hat{p}}_{r}=p_{r}\left(\hat{\xi },\hat{\pi }\right)$) (D'Elia and Piccolo, 2005). The dissimilarity index (Diss), normalised in [0,1], is explicated in Eq. (2):

(2) $$Diss=\frac{1}{2}\overset{m}{\underset{r=1}{\sum }}|f_{r}-p_{r}\left(\hat{\xi },\hat{\pi }\right)|$$

If Diss<0.1, the estimated CUB model is associated with a good fitting (Iannario, 2009).

The estimated feeling ($1-\hat{\xi }$) and uncertainty ($1-\hat{\pi }$) might be usefully represented in the parameter space (unit square): in vertical axis, values of $\left(1-\hat{\xi }\right)$ close to 1 indicate a high degree of liking with respect to the analysed item; in horizontal axis, values of $\left(1-\hat{\pi }\right)$ close to 1 suggest a propensity of respondents to make a random choice.

#### 2.2.2 The CUB(p, q) models

The CUB models in Eq. (1) may be also estimated conditional to the influence of *p* and *q* covariates on feeling and uncertainty respectively, related to the *i*-th respondent. For any *i*-th respondent, the CUB(*p*, *q*) models are defined as a stochastic process (Eqs. (3) and (4)):

(3) $$\mathrm{Pr}\left(R_{i}=r|y_{i};w_{i}\right)=\pi_{i}\left(\begin{array}{l} m-1\\ r-1 \end{array}\right)\xi_{i}^{m-r}{\left(1-\xi_{i}\right)}^{r-1}+\left(1-\pi_{i}\right)\left(\frac{1}{m}\right),\;r=1,2,\ldots ,m\;\text{and}\;i=1,2,\ldots ,n$$

with

(4) $${\text{ξ}}_{\text{i}}={\text{ξ}}_{\text{i}}\left(\gamma \right)=\frac{1}{1+{\text{e}}^{-w_{\text{i}}\gamma }}\;\text{and}\;\pi_{i}={\text{π}}_{\text{i}}\left(\beta \right)=\frac{1}{1+e^{-y_{i}\beta }},\;r=1,2,\ldots ,m\;\text{and}\;i=1,2,\ldots ,n$$

where $R_{i}$ is the random variable for the *i*-th respondent, *r* is the rating, m is the number of categories of the evaluation scale; $y_{i}={\left(1,y_{i1},\ldots ,y_{1p}\right)}^{\text{′}}$ and $w_{\text{i}}={\left(1,{\text{w}}_{\text{i}1},\ldots ,{\text{w}}_{\text{iq}}\right)}^{\text{′}}$ are the vectors of the *p* and *q* covariates related to feeling ($\xi_{i}$) and uncertainty ($\pi_{i}$) of the *i*-th respondent; β and γ are parameters referred to uncertainty and feeling.

According to the equation in (4), for k ranging between 1 and *m*, if $w_{k}$ ($y_{k}$) increases positively, there is an increase in feeling (uncertainty) if $\gamma_{k}<0$ (${\text{β}}_{\text{k}}<0$), and a decrease in feeling (uncertainty) if ${\text{γ}}_{\text{k}}>0$ (${\text{β}}_{\text{k}}>0$) (Iannario and Piccolo, 2012).

The CUB models are estimated through a maximum likelihood (ML) estimation, via Expectation-Maximization (EM) algorithm. To assess the adequacy of sample size for deriving acceptable inferential results, when the number (*m*) of values on Likert scale is fixed, the ratio *k* = *n*/*m* (where *n* is the number of respondents) is used. Generally, the bias decreases when k becomes large, for both the parameters (D'Elia 2003; D'Elia and Piccolo, 2005).

### 2.3 Statistical analyses

In order to investigate consumers' perception of organic food, the CUB(0, 0) models in Eq. (1) are fitted on the attributes ‘healthiness’, ‘safety’, and ‘sustainability’ of organic food.

The CUB(*p, q*) models in Eqs. (3) and (4) are fitted on the attributes ‘healthiness’, ‘safety’, and ‘sustainability’, in order to examine if and how the perception of organic food is affected by (i) consumers' socio-demographic profile and (ii) the presence of specific labels.

The hypothesis (i) is tested using socio-demographic characteristics of respondents as covariates, arranged by two levels: ‘gender’ (male = 0; female = 1), ‘educational level’ (primary and middle school = 0; upper secondary school and bachelor/master's degree = 1), ‘financial situation’ (difficult, modest, discreet = 0; good, very good = 1), and ‘weekly spending for food’ (<150 € = 0; >150 € = 1) (DiStefano et al., 2021). We selected these four variables on the basis of evidence from previous research on the role that socio-demographics plays in profiling consumers who are concerned about environmental, health, and safety issues (e.g., Diamantopoulos et al., 2003; Li and Kallas, 2021). While empirical studies tend to profile consumers through different socio-demographic variables, gender, education levels, financial situation (thus spending for food) are the most explicative information (e.g., Solomon et al., 2006; Ghvanidze et al., 2016). We also analysed the effect of age covariate considering five groups of respondents (”18–25”, “26–35”, “36–45”, “46–55”, “above 55” years old).

The hypothesis (ii) is tested using items related to specific labels (‘label info’, ‘health claims’, ‘quality label’, ‘organic label’, ‘environmental label’) as covariates, arranged by two levels of importance: ‘low and medium’ for scores from 1 to 4 of the Likert scale points, ‘high’ for scores from 5 to 7 of the Likert scale points (e.g., Piccolo and Simone, 2019).

The ratio *k* = 96 (with *k* > 30) suggests the asymptotical unbiasedness of ML estimators providing the appropriateness of sample size.

Inferential issues, fully specified in Piccolo (2006), are implemented in package CUB 3.0, available in R environment (Iannario et al., 2020). The 5% level is adopted as statistically significant for all the analyses performed in this study.

## 3 Results and discussion

### 3.1 Consumers’ perception of organic food

The results of the CUB models, reported in Table 3, show the consumers’ perception of organic food in terms of healthiness, safety, and environmental sustainability. The estimated values for feeling ($1-\hat{\xi }$) and uncertainty ($1-\hat{\pi }$) are large and statistically significant at 5% level for all attributes. The dissimilarity index is always lower than 0.1, indicating a good fit of the estimated CUB models.

**Table 3:** Consumers’ perception of organic food: estimation of CUB(0,0) models and dissimilarity index.

Attributes	Feeling ($1-\hat{\xi }$)	Uncertainty ($1-\hat{\pi }$)	Dissimilarity index
Healthiness	0.83∗	0.74∗	0.05
	(0.03)	(0.04)	
Safety	0.92∗	0.85∗	0.02
	(0.03)	(0.03)	
Sustainability	0.82∗	0.78∗	0.06
	(0.03)	(0.04)	

Standard errors are in parentheses; ∗ indicates *p-values* < 0.05.

**Figure 2:**
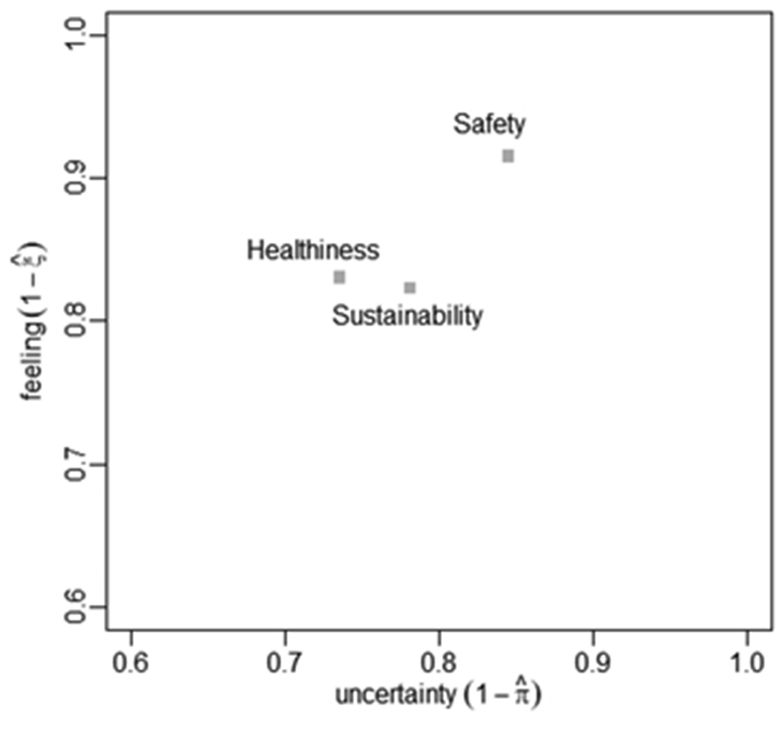
Consumers' perception of organic food: representation in the parametric space of estimated feeling and uncertainty.

The parametric space in Figure 2 represents estimated results in terms of latent components (feeling and uncertainty). The high levels of feeling suggest that consumers tend to perceive organic food as healthy, safe, and environmentally sustainable. However, the high levels of uncertainty reveal a marked heterogeneity among consumers’ opinions. The results might depend on the sample composition. A high percentage of respondents are younger and better educated consumers and they tend to manifest a strong feeling for environmental goods and socially desirable products, such as organic food, and are more prone to read labels (e.g., Ghvanidze et al., 2016; Li and Kallas, 2021). In fact, as reported in Table 4, the effect of the age is significant for younger groups of respondents. Accordingly, the literature reports a significant impact of age on the perception of organic food: this is likely to be associated to a different lifestyle (e.g., Padel and Foster, 2005). Accordingly, the stakeholders of the organic sector (e.g., producers, processors, marketers) should set this segment of consumers as a target to expand their market share.

**Table 4:** Consumers’ perception of organic food by age: estimation of CUB (0,1) models.

Attributes	Feeling ($1-\hat{\xi }$)					Uncertainty ($1-\hat{\text{π}}$)
	Age					
	$({\hat{\gamma }}_{0}^{\text{§}}$)	(${\hat{\text{γ}}}_{1:26-35}$)	(${\hat{\text{γ}}}_{1:36-45}$)	(${\hat{\text{γ}}}_{1:46-55}$)	(${\hat{\text{γ}}}_{1:55+}$)	
Healthiness	-1.97∗	0.94∗	-0.12	-0.17	0.71	0.73∗
	(0.41)	0.47	0.57	0.74	0.58	0.04
Safety	-2.94∗	1.67∗	0.54	3.85∗	1.78	0.77∗
	(0.65)	0.71	0.75	0.81	0.78	0.037
Sustainability	2.24∗	1.53∗	0.15	2.68∗	0.85	0.72∗
	(0.45)	(0.49)	(0.54)	(0.68)	(0.66)	(0.04)

Standard errors are in parentheses; ∗ indicates *p-values* < 0.05. Levels of importance are assumed for the covariate ‘age’: 18–25 (^§^ reference category), 26–35, 36–45,46-55, 55+.

The attribute ‘safety’ has the highest feeling (0.92), but also the highest uncertainty (0.85) (Table 3). As also demonstrated in literature (e.g., Lee and Yun, 2015), consumers tend to perceive organic products as food free from harmful contents, such as GMOs. The attributes ‘healthiness’ and ‘environmental sustainability’ are less important in terms of feeling (0.83 and 0.82, respectively), but they present lower levels of uncertainty (0.74 and 0.78, respectively) (Table 3). This is consistent with previous studies reporting that the attribute organic influences consumers' perception of healthfulness and environmental sustainability of food, for example with respect to methods of production (Lee et al., 2013; Prada et al., 2016). In addition, the higher uncertainty for environmental sustainability than healthiness may depend on the fact that, while health concerns are directly related to the health condition of individuals, environmental matters are impersonal drivers highly dependent on respondents' awareness (Ghvanidze et al., 2016).

Overall, our results highlight that consumers tend to associate organic food with the idea of safety, more than of healthiness or environmental sustainability. As found in Załęcka et al. (2014), organic food tends to contain fewer pesticide residues and selected health-related compounds, but the health relevance for consumers is not clear yet. Producers, processors and marketers in the organic sector should work towards the communication of safety associated to organic products. Consumers are becoming more concerned about the safety of the products they buy and consume. Frequently, this concern is due to an information gap between producers and (that are better informed than) consumers (Santeramo and Lamonaca, 2021). If organic products convey the idea of safe food, the gain would be in the reduction of the information gap to the benefits of both consumers, in terms of more aware consumption, and producers, in terms of higher retail sales, premium prices, market share.

### 3.2 Consumers’ profile as drivers of perception of organic food

The CUB models are estimated using as covariates four socio-demographic variables, i.e., ‘gender’, ‘educational level’, ‘financial situation’, ‘weekly spending for food’^7^. Not statistically significant relationships are found between the dependent variables feeling and uncertainty and the covariates ‘educational level’, ‘financial situation’, ‘weekly spending for food’^8^. Differently, a statistically significant effect of ‘gender’ is found for feeling but not for uncertainty, indicating that males and females behave differently in scoring the degree of feeling of attributes (i.e., healthiness, safety, environmental sustainability). The results are consistent with previous findings, demonstrating how gender is determinant in influencing consumers' perception of organic food (e.g., Hsu et al., 2012; Hsu and Chen, 2014). Indeed, the gender dimension is a relevant source of heterogeneity explaining how consumers judge sustainable food products (Li and Kallas, 2021).

The results of CUB models estimated using ‘gender’ as covariate for feeling are shown in Table 5. The estimated values for uncertainty ($1-\hat{\pi }$) are large and statistically significant at 5% level for all attributes. The estimated parameters for males (${\hat{\gamma }}_{0}$) are significant negative for all attributes, whereas the estimated parameters for females (${\hat{\gamma }}_{1}$) are significant positive for ‘healthiness’ and ‘safety’, and significant negative for ‘sustainability’.

**Table 5:** Consumers’ perception of organic food by gender: estimation of CUB (0,1) models.

Attributes	Feeling ($1-\hat{\xi }$)		Uncertainty ($1-\hat{\text{π}}$)
	Male (${\hat{\gamma }}_{0}$)	Female (${\hat{\text{γ}}}_{1}$)	
Healthiness	-2.52∗	0.70∗	0.73∗
	(0.51)	(0.33)	(0.04)
Safety	-4.53∗	1.84∗	0.81∗
	(0.86)	(0.53)	(0.03)
Sustainability	-2.81∗	-0.98∗	0.77∗
	(0.59)	(0.37)	(0.04)

Standard errors are in parentheses; ∗ indicates *p-values* < 0.05. Two level of importance (0 = male, 1 = female) are assumed for the covariate ‘gender’.

**Figure 3:**
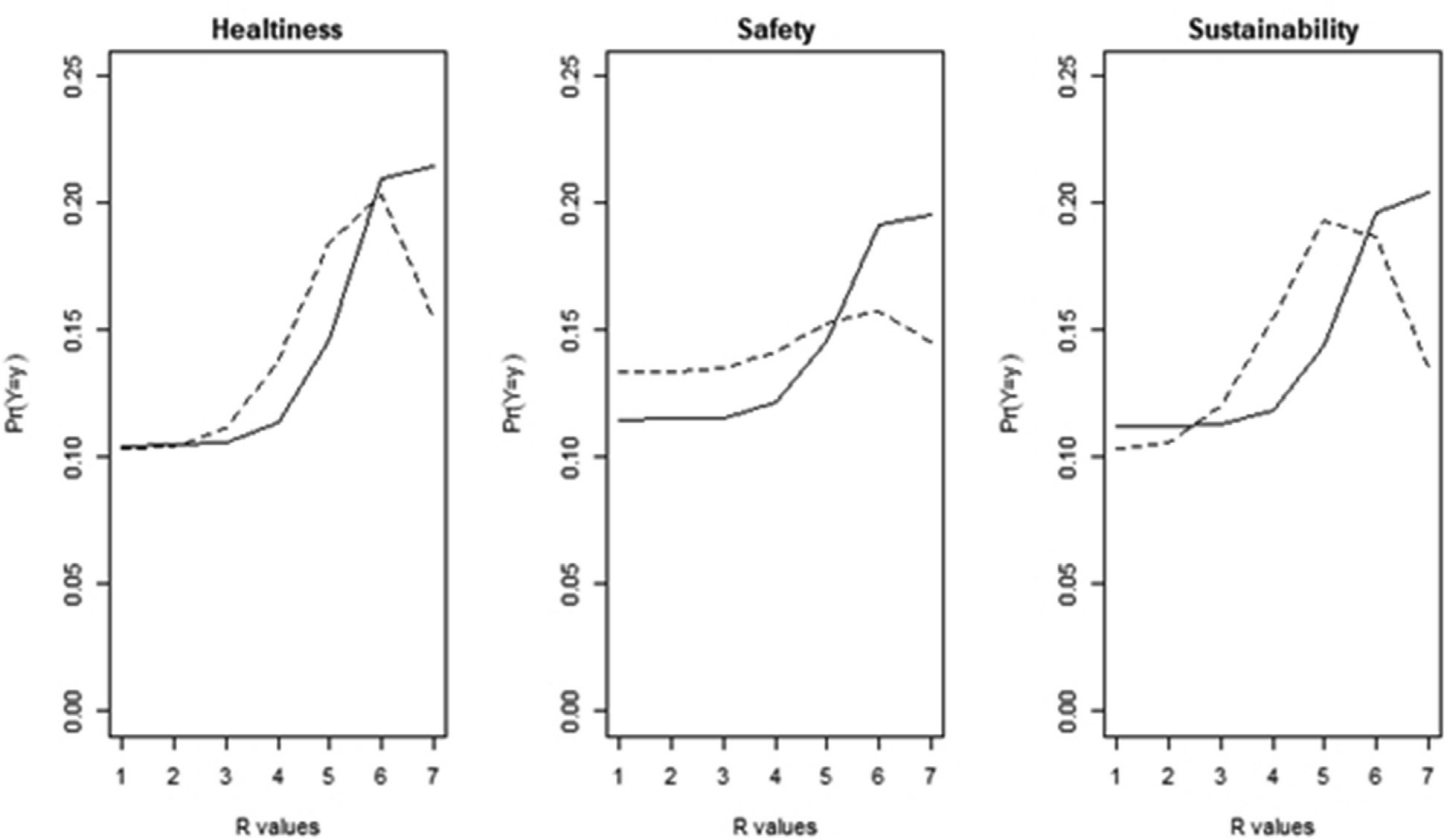
Consumers' perception of organic food by gender: estimated CUB distributions. Notes: Patterns of probabilities in figure are discrete probability distributions. The estimated distributions are in solid line for males and in dashed line for females.

The results reveal that males have a higher feeling than females for organic food. Females perceive organic food as sustainable, but to a lower extent than males. Reviewing evidence on the role of socio-demographics in profiling green consumers, Diamantopoulos et al. (2003) highlight that males tend to have higher and better knowledge about green issues than females. The estimated distributions for males and females are plotted and compared in Figure 3 for each attribute.

The distributions for males and females are right shifted for each attribute, highlighting a propensity to choose high levels of feeling/liking. While males are quite similar in rating each attribute, females show differences in ordinal distributions for ‘safety’ as compared to ‘healthiness’ and ‘sustainability’. This may be due to differences between males and females in terms of concerns about food safety and adoption of conscious behaviours (Hunter et al., 2004; Ghvanidze et al., 2016). Findings suggest that the perception of organic food in terms of estimated feeling/liking is lower for females than for males. As shown in previous studies (e.g., Hsu et al., 2012; Hsu and Chen, 2014), the perception of organic food is likely to differ between males and females. The fact that males appreciate the organic attribute more than females may be due to cultural differences and consumption habits in different countries. For instance, D'Amico et al. (2016) found that the organic attribute is better perceived by males in Italy and Galati et al. (2019) note that in Spain the perception of the organic attribute is higher among females.

Moreover, the perception of organic food shows no statistically significant response to consumers’ socio-demographic characteristics; accordingly, we cannot conclude on the relationships between perception of organic food and educational level, financial situation, or weekly spending for food. Our results may be due to the particular composition of our sample, for example in terms of age and number of children per household. Younger consumers may not have children yet, and income might not be yet a concern; this would also affect the weekly spending for food. In fact, a common finding in literature is the significant impact of age (e.g., Padel and Foster, 2005), number of children (e.g., Tiffin and Arnoult, 2010) and income levels on preferences for organic products.

### 3.3 Labels as drivers of consumers’ perception of organic food

The CUB models are estimated using different types of labels as covariates for feeling; results are in Table 6. None of the covariates are statistically significant with respect to uncertainty, thus results have been omitted.

**Table 6:** Consumers’ perception of organic food by label: estimation of CUB (0,1) models.

Attribute	Covariates	Feeling ($1-\hat{\xi }$)		Uncertainty ($1-\hat{\text{π}}$)
		Low and medium (${\hat{\gamma }}_{0}$)	High (${\hat{\text{γ}}}_{1}$)	
Healthiness	Label info	4.79∗	-3.15∗	0.52∗
		(0.44)	(0.24)	(0.04)
	Health claims	4.51∗	-3.08∗	0.57
		(0.44)	(0.24)	(0.04)
	Quality label	4.85∗	-3.18∗	0.54∗
		(0.44)	(0.24)	(0.04)
	Organic label	4.22∗	-2.89∗	0.46∗
		(0.35)	(0.20)	(0.04)
	Environmental label	4.37∗	-3.05∗	0.64∗
		(0.60)	(0.34)	(0.04)
Safety	Label info	5.53∗	-3.69∗	0.62∗
		(0.48)	(0.29)	(0.04)
	Health claims	4.47∗	-3.17∗	0.58
		(0.48)	(0.29)	(0.04)
	Quality label	5.52∗	-3.65∗	0.60∗
		(0.47)	(0.29)	(0.04)
	Organic label	4.91∗	-3.37∗	0.56∗
		(0.40)	(0.25)	(0.04)
	Environmental label	4.87∗	-3.44	0.67∗
		(0.51)	(0.32)	(0.04)
Sustainability	Label info	5.03∗	-3.22	0.58∗
		(0.48)	(0.27)	(0.04)
	Health claims	4.00∗	-2.79∗	0.58∗
		(0.48)	(0.27)	(0.04)
	Quality label	4.91∗	-3.16∗	0.56∗
		(0.47)	(0.26)	(0.04)
	Organic label	4.28∗	-2.91∗	0.54∗
		(0.42)	(0.25)	(0.04)
	Environmental label	4.22∗	-2.93∗	0.62∗
		(0.54)	(0.31)	(0.04)

Standard errors are in parentheses; ∗ indicates *p-values* < 0.05. Two level of importance (0 = low and medium, 1 = high) are assumed for each covariate.

The estimated parameters for ‘low and medium importance’ (${\hat{\gamma }}_{0}$) are significant positive, whereas the estimated parameters for ‘high importance’ (${\hat{\gamma }}_{1}$) are significant negative in each case. Findings suggest that the higher the level of importance attributed by consumers to the presence of specific labels on organic food, the higher the feeling related to the perception of healthiness, safety, and environmental sustainability. In particular, findings reveal that consumers tend to perceive organic food as healthier based on product fact tables and the presence of health claims, which show the highest levels of feeling. Similarly, the perception to consume safe and environmentally sustainable food increases with the presence of a detailed facts table and the organic label (i.e., items with the greater level of feeling). The presence of specific labels also reduces the uncertainty related to the perception of organic food (the estimated values for uncertainty range between 0.46 and 0.67).

**Figure 4:**
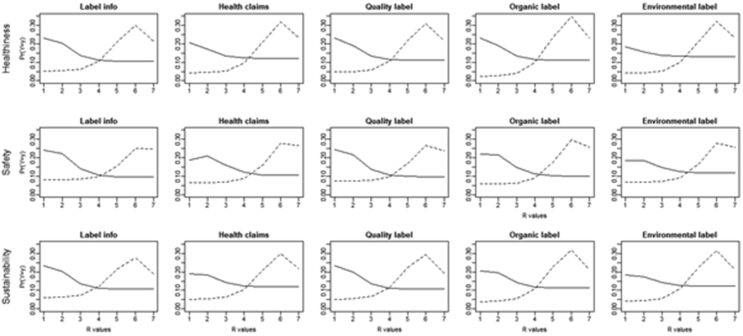
Consumers' perception of organic food by label: estimated CUB distributions. Notes: Patterns of probabilities in figure are discrete probability distributions. The estimated distributions are in solid line for low and medium importance and in dashed line for high importance.

The estimated distributions for low and high importance (Figure 4) are clearly different for each combination attribute-covariate. The distributions of consumers that attribute high level of importance are always right shifted.

In a nutshell, consumers that attribute high importance to the presence of specific labels tend to perceive organic food as healthier, safer, and more environmentally sustainable. These findings are in line with previous studies which suggest that modern consumers are aware of the impacts that their buying behaviour may cause on environmental and socio-economic life’ aspects (Lee and Yun, 2015), and tend to choose healthy and environmentally friendly food products (Moser, 2016). In particular, a detailed facts table is the most influencing information for consumers. A plausible explanation is that nowadays consumers are more familiar with information on food labels and are used to consult them (Ghvanidze et al., 2016).

## 4 Conclusions

This study investigated the perceptions of a panel of consumer about quality of organic food, in terms of environmental sustainability, safety and healthiness. By adopting an approach based on CUB models, this study examined the latent attitude (e.g., Daadi and Latacz-Lohmann, 2021) of consumers towards organic food. In particular, it investigated how and to what extent perceived quality of organic food is influenced by the socio-demographic profile of consumers and the presence of qualitative information on products’ label.

The results suggested that consumers tend to perceive organic food as healthy, safe, and environmentally sustainable. In particular, consumers are less uncertain in judging healthiness and environmental sustainability of organic food. As shown in Lee et al. (2013) and Prada et al. (2016), healthiness and environmental sustainability are inherent characteristics of organic food. However, differences in consumers’ perception exist between males and females, as also shown in previous studies on the issue (e.g., Galati et al., 2019; Li and Kallas, 2021). Organic food tends to be well perceived by males, but females are more concerned about sustainability of organic food. The presence of specific labels contributes to increase the perception of organic food as healthy, safe, and environmentally sustainable. Detailed information on labels is the most influencing attribute for consumers; the higher the detail, the higher the feeling and the lower the uncertainty of consumers related to the perception of organic food. Consumers are more familiar with information on food labels and are used to consult them (Ghvanidze et al., 2016).

Future research may be devoted to deepening on differences in the perceptions of organic food between users and non-users. The results may be related to the particular composition of the sample, for example in terms of age and number of children per household. In fact, a common finding in literature is the significant impact of age, number of children and income levels on preferences for organic products (e.g., Tiffin and Arnoult, 2010). Future research may expand the findings by investigating heterogeneity in the perception of organic food on a class (segment) level.

Findings from this analysis may support marketers and producers by suggesting which information is most valued by customers. Producers may consider strategies to improve the image of their organic production by including and communicating messages of healthiness via detailed information on labels or health claims, absence of harmful ingredients through quality and organic labels, and sustainability by means of environmental labels. Understanding consumer perceptions enables marketers to propose tailor-made strategies to successfully communicate benefits of organic food. This is of particular relevance in a framework where producers have to choose among several labels which potentially affect consumers’ decision-making process. In this regard, the dialogue between producers and consumers may contribute to achieve the match between production and consumption of organic food. Relevant evidence of this analysis is that consumers are more confident with healthiness and sustainability of organic food. The increasing concern of consumers about health and green issues has to be encouraging for producers and processors of organic food that, according to their inherent mission and vision, are committed to preserve both the health and the environment. While health and green requirements are becoming fundamental for customer satisfaction, organic products should also be improved in terms of competitiveness with proper labels. Food labels increase the perception of organic food as healthy, safe, and environmentally sustainable: the more detailed the information on food labels, the greater the confidence of consumers. The use of labels conveying information of the healthiness, safety, and environmental sustainability of organic products would allow organic producers and processors to better position their products in an ever-growing organic market. In this context, the role of policymakers is of utmost importance. Policy actions should be oriented towards the recognition of locally focused quality assurance systems (e.g., Participatory Guarantee Systems) and third-party certifications. By supporting these schemes through national organic policies and regulations, policymakers may improve the access to organic markets for small-scale producers, increase the awareness and engagement among consumers, promote short supply chains and local market development (IFOAM - International Federation of Organic Agriculture Movements, 2018).

## Declarations

### Author contribution statement

Emilia Lamonaca: Conceived and designed the experiments; Performed the experiments; Analyzed and interpreted the data; Wrote the paper.

Barbara Cafarelli; Crescenza Calculli: Performed the experiments; Analyzed and interpreted the data; Contributed reagents, materials, analysis tools or data.

Caterina Tricase: Conceived and designed the experiments.

### Funding statement

B. Cafarelli and C. Calculli were supported by the PRIN2015 project EphaStat – Environmental processes and human activities: capturing their interactions via statistical methods, funded by the Italian Ministry of University and Research (MIUR).

### Data availability statement

Data will be made available on request.

### Declaration of interests statement

The authors declare no conflict of interest.

### Additional information

No additional information is available for this paper.

## Figures and Tables

**Table:** 

□	□	□	□	□	□	□
1	2	3	4	5	6	7

**Table:** 

□	□	□	□	□	□	□
1	2	3	4	5	6	7

**Table:** 

□	□	□	□	□	□	□
1	2	3	4	5	6	7

**Table:** 

□	□	□	□	□	□	□
1	2	3	4	5	6	7

**Table:** 

□	□	□	□	□	□	□
1	2	3	4	5	6	7

**Table:** 

□	□	□	□	□	□	□
1	2	3	4	5	6	7

**Table:** 

□	□	□	□	□	□	□
1	2	3	4	5	6	7

**Table:** 

□	□	□	□	□	□	□
1	2	3	4	5	6	7

## Appendix A

### SURVEY ON CONSUMPTION OF ORGANIC PRODUCTS

Section I: SOCIO-DEMOGRAPHIC CHARACTERISTICS.

**1.**Gender:

Female
Male

**2.**Age (years)

**3.**Number of household members

**4.**Education:

Primary school
Middle school
Upper secondary school
Bachelor/Master's degree or equivalent

**5.**Weekly spending for food (€)

Section II: ATTRIBUTES OF ORGANIC FOOD.

**6.**Please, indicate your opinion on the following statements using a scale from 1 (completely disagree) to 7 (completely agree):

**a.** Organic food is healthier

**b.** Organic food is safer

**c.** Organic food is environmentally sustainable

Section III: LABELS.

7. Please, indicate the importance of the following labels in your buying behavior using a scale from 1 (completely disagree) to 7 (completely agree):

**a.** Label info

**b.** Health claims

**c.** Quality label

**d.** Organic label

**e.** Environmental label

## Footnotes

1 Founded in 1972, IFOAM – Organics International is a membership-based organisation working to bring true sustainability to agriculture across the globe.

2 The FAO Glossary on Organic Agriculture is available at the following link.

3 The Codex Alimentarius is a collection of internationally adopted food standards, guidelines, codes of practice and other recommendations, whose aim is to protect the health of consumers and ensure fair practices in the food trade. It is prepared by the Codex Alimentarius Commission, an intergovernmental body with over 170 members, within the framework of the Joint FAO/WHO Food Standards Programme established by the FAO and the World Health Organisation (WHO). More details are available at www.fao.org.

4 More details at www.fao.org.

5 More details at www.efsa.europa.eu.

6 The use of these models is proposed to analyse consumers' perceptions in terms of two latent components, feeling and uncertainty. The suggested approach is suitable for such analyses that involve consumers perception. The application of this kind of models does not require the performance of a reproducibility test.

7 Recall that the choice of the socio-demographic variables is based on evidence from previous research indicating that gender, education, income (e.g., financial situation, spending for food) tend to affect the most how consumers perceive environmental, health, and safety information about food products (e.g., Diamantopoulos et al., 2003; Li and Kallas, 2021).

8 The results, omitted for brevity, are available upon request.
